# Targeted delivery of rhodopsin’s assembled core is required for outer segment extension in mouse rod photoreceptors

**DOI:** 10.1016/j.jbc.2025.111106

**Published:** 2025-12-23

**Authors:** Jorge Y. Martínez-Márquez, Sandy Hua, Andreea M. Beu, Christopher B. Stein, Jillian N. Pearring

**Affiliations:** 1 Department of Ophthalmology and Visual Science, University of Michigan, Ann Arbor, Michigan, USA; 2 Department of Psychology, Hunter College, New York, New York, USA; 3 Department of Cell and Developmental Biology, University of Michigan, Ann Arbor, Michigan, USA

**Keywords:** outer segment, photoreceptors, retina, rhodopsin, membrane traffic, rods

## Abstract

Vertebrate vision in dim-light environments is initiated by rod photoreceptor cells that express the photopigment rhodopsin, a G protein–coupled receptor. To ensure efficient light capture, rhodopsin is densely packed into hundreds of tightly stacked membrane discs within the rod-shaped outer segment (OS) compartment. Along with its role in eliciting the visual response, rhodopsin serves as a building block necessary for proper OS formation and a trafficking guide for a few OS resident membrane proteins. An interesting aspect of rod homeostasis is that mutations that affect the localization of rhodopsin to the OS result in photoreceptor degeneration. In this study, we focus on determining the properties of rhodopsin’s cytosolic C terminus required for either proper OS trafficking or the capacity to extend the rudimentary OS in rhodopsin KO rods. We find that the well-described C-terminal QVAPA OS targeting motif also plays a role in endoplasmic reticulum exit and is necessary for elongation of the OS compartment. We identify that rhodopsin’s core, helix-8, CC anchor, and QVAPA targeting motif are the minimal requirements to extend the rudimentary OS in rhodopsin KO rods. Our findings provide useful insights into rhodopsin’s molecular features needed for OS delivery and subsequent elongation of this membrane-rich compartment.

Retinal photoreceptor cells detect light in their outer segment (OS) organelle, a large modified primary cilium stacked with hundreds of disc-shaped membranes. In rod photoreceptors, disc membranes are packed with rhodopsin, a G protein-coupled receptor (GPCR) that initiates the visual response to light (1). Rhodopsin is synthesized and trafficked through the endoplasmic reticulum (ER) and Golgi complex before being delivered *via* intracellular vesicle traffic to the apical membrane of the inner segment (2, 3, 4, 5). From here, rhodopsin travels through the connecting cilium to the OS, where it helps drive disc membrane formation. Currently, more than 150 rhodopsin variants have been identified in human patients with retinitis pigmentosa, accounting for 30% to 40% of all cases (6, 7). Mutations that cause a defect in the trafficking and delivery of rhodopsin to the OS result in some of the most severe cases of autosomal dominant retinitis pigmentosa (8, 9, 10). Understanding how rhodopsin is trafficked to the OS is imperative to developing future therapeutic targets for these patients.

Rhodopsin is a seven-transmembrane (TM) domain protein with an extracellular N terminus and a cytosolic C terminus. Rhodopsin’s extracellular N terminus is glycosylated at two asparagine residues (N2 and N15) (11, 12). Rhodopsin’s seven-TM helical core forms the binding pocket for the chromophore, 11-*cis*-retinal, which isomerizes to all-*trans*-retinal upon photon energy transfer, inducing rhodopsin conformational changes and activation (13, 14). Rhodopsin’s core is followed by an eighth amphipathic α-helix [helix-8] and two palmitoylated cysteines (CC anchor), which position helix-8 parallel to the membrane. Rhodopsin’s helix-8 has been implicated in binding to 11-*cis*-retinal, G-protein activation, and protein folding (15).

A recent study showed that key residues in rhodopsin’s helix-8 form hydrophobic interactions with TM1 to stabilize the core structure of rhodopsin (16). This study found that mutants disrupting these hydrophobic interactions caused mislocalization of rhodopsin to the inner segment. Following the CC anchor, rhodopsin ends with a largely unstructured string of amino acids. This intracellular tail contains multiple sites for post-translational modification, which are used to recruit proteins in the visual signaling pathway, and encode trafficking signals essential for the delivery of rhodopsin to the OS (17, 18, 19, 20, 21).

Membrane proteins can ensure proper delivery to their site of action by encoding a targeting signal. Rhodopsin contains a C-terminal QVAPA targeting motif, which is both necessary and sufficient for OS delivery (21, 22). Interestingly, untargeted membrane proteins exogenously expressed in rods localize throughout the plasma membrane and the OS. This localization pattern, previously coined the “default” trafficking pathway (23, 24, 25), likely occurs because the OS is a membrane-rich organelle that does not undergo typical protein turnover. Instead, OS membranes undergo renewal by adding new discs at the base and phagocytosing old discs from the tip by the retinal pigmented epithelium (26, 27). Ongoing disc formation places a high demand on membrane transport toward the OS, thought to drive the “default” pathway. Importantly, these data show that a specific targeting signal is required to prevent membrane proteins from localizing to the OS.

Rhodopsin is an integral building block of the OS. Rhodopsin KO (RhoKO) rods extend small, shapeless ciliary compartments filled with disorganized membranes that carry the full complement of OS resident proteins (28, 29, 30, 31). Failure to elaborate a proper OS leads to the rapid degeneration of rod photoreceptors in RhoKO mice (28, 30). It was previously shown that a rhodopsin Q334Ter mutant mislocalizes to the inner segment, even preventing proper localization of endogenous rhodopsin, and was unable to properly elongate rod OSs or rescue rod degeneration in RhoKO mice (32). These results suggest that specific targeting and efficient delivery of rhodopsin are needed for OS formation and healthy rods. However, a thorough understanding of rhodopsin’s molecular features is required to elucidate the interplay between targeted delivery and subsequent OS elongation.

## Results

### Pipeline to analyze membrane protein localization profiles of electroporated rod photoreceptors

We employed an *in vivo* electroporation technique to understand the localization patterns of FLAG-tagged rhodopsin constructs in mouse rod photoreceptors. We began by establishing a standardized method to quantify the localization of a particular construct to analyze data across animals and compare localization profiles from different constructs. To do this, we measured the fluorescence intensity present in the OS compartment, which was delineated by a soluble mCherry transfection marker coelectroporated with all our constructs. The mCherry signal is primarily found in the inner segment, nuclear, and synaptic compartments because the membrane-rich OS has very little cytosol. For a given image, the OS fluorescence signal of a particular construct was then divided by its total fluorescence in all rod compartments, which results in an OS/total intensity value for each image, where 1.0 is fully localized to the OS, and 0.0 is fully excluded from the OS.

**Figure 1:**
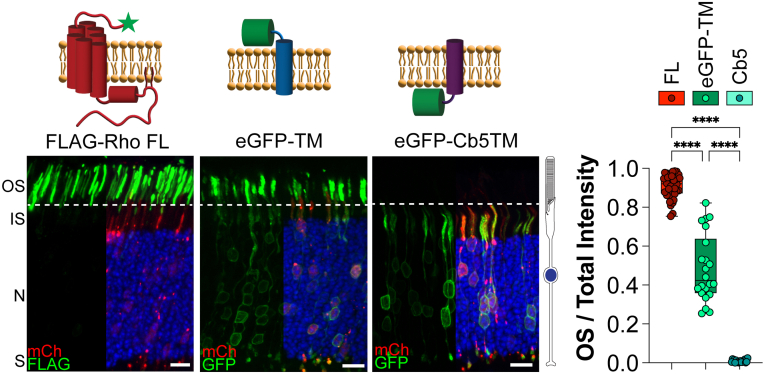
**Localization profile of OS targeted, untargeted, and endoplasmic reticulum (ER)–retained membrane proteins in WT mouse rods.** The following constructs were electroporated into WT mouse rods: FLAG-tagged full-length rhodopsin (Rho FL), eGFP-tagged transmembrane (TM) segment 1 from mGluR1 (eGFP-TM), eGFP-tagged TM domain from microsomal cytochrome b5 (eGFP-Cb5TM). FLAG staining or GFP (*green*), mCherry (*red*) labels transfected rod cells, and DAPI (*blue*) was used to counterstain nuclei. The scale bar represents 10 μm. To the *right* is a *cartoon* of a rod photoreceptor. The bar graph shows the quotient between the OS signal over the total signal for each construct. Each point represents a single image. FL n = 12, 38 images; eGFP-TM n = 7, 22 images; and Cb5 n = 6, 19 images. Only half of the DAPI and mCherry fluorescence is shown in the images to provide an unobstructed view of the green channel’s fluorescence. A *cartoon diagram* for each construct is shown on *top* of the representative image, with the FLAG tag depicted with a *green star*. All *p* values can be found in Figure S1. DAPI, 4′,6-diamidino-2-phenylindole; eGFP, enhanced GFP; IS, inner segment; N, outer nuclear layer; OS, outer segment; S, synapses.

We initially established the localization profile for full-length FLAG-tagged rhodopsin (FLAG-Rho FL), which resulted in a mean OS/total intensity value of 0.899 ± 0.063 (Fig. 1). To establish the localization profile for an untargeted membrane protein, we used an enhanced GFP (eGFP)-mGluR1_TM1_ construct (N-terminal eGFP fused to the first TM domain of mGluR1), previously shown to reach the OS through “default” trafficking in both WT mouse and frog rods (23, 25). The mean OS/total intensity value for eGFP-mGluR1_TM1_ was 0.510 ± 0.149, supporting that untargeted constructs have an equal distribution between the OS and plasma membrane surrounding the rod cell body (Fig. 1). Finally, to determine the localization profile for a membrane protein that is retained in the ER, we chose to express an eGFP-tagged TM domain from a microsomal cytochrome b5 (eGFP-Cb5TM). This construct was previously shown to localize within the ER of frog rods (23) and, therefore, is not present within the OS compartment. The eGFP-Cb5TM construct resulted in a mean OS/total intensity value of 0.007 ± 0.005 (Fig. 1). These data establish the localization profile for targeted, untargeted, or ER-retained membrane constructs in WT rods.

### Determining the localization profiles of C-terminally truncated rhodopsin in WT mouse rods

**Figure 2:**
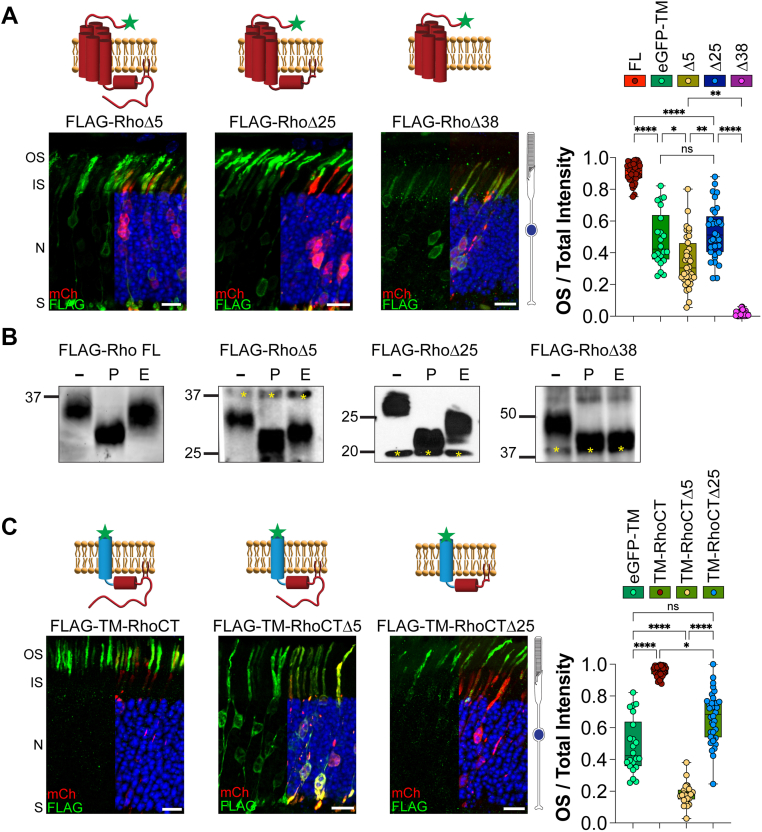
**Rhodopsin C-terminal truncations reveal unique localization profiles in WT mouse rods.***A*, the following FLAG-tagged rhodopsin constructs were electroporated into WT mouse rods: rhodopsin lacking its final five amino acids (RhoΔ5), 25 amino acids (RhoΔ25), or 38 amino acids (RhoΔ38). Δ5 n = 9, 35 images; Δ25 n = 10, 33 images; and Δ38 n = 6, 27 images. *B*, FLAG-tagged rhodopsin constructs immunoprecipitated from electroporated RhoKO mouse retinal lysates were treated with PNGase F or endo H and analyzed by Western blot. Untreated lysates (−), PNGase F (P), endo H (E). Resistance to endo H treatment indicates endoplasmic reticulum (ER) exit and normal processing through the conventional secretory pathway for FLAG-Rho, FLAG-RhoΔ5, and FLAG-RhoΔ25. Sensitivity to both PNGase F and endo H treatments shows increased electrophoretic mobility for FLAG-RhoΔ38, indicative of retention within the ER. Due to the reduced molecular weight of FLAG-RhoΔ38, the rhodopsin dimer (∼48 kDa) that persists under denaturing conditions was used to track the deglycosylation status of FLAG-RhoΔ38. *Yellow asterisks* indicate nonspecific bands. *C*, WT mouse rods were electroporated with FLAG-tagged, single-pass transmembrane domain fused to intact C-terminal cytosolic tail of rhodopsin (TM-RhoCT), or rhodopsin’s C-terminal tail lacking the final five amino acids (TM-RhoCTΔ5) or the final 25 amino acids (TM-RhoCTΔ25). TM-RhoCT n = 4, 41 images; TM-RhoCTΔ5 n = 6, 24 images; and TM-RhoCTΔ25 n = 5, 36 images. Bar graphs show the quotient between the OS signal over the total signal for each construct and are plotted next to the untargeted eGFP-TM from Figure 1. FLAG staining (*green*), mCherry (*red*) labels transfected rod cells, and DAPI (*blue*) was used to counterstain nuclei. The scale bar represents 10 μm. DAPI, 4′,6-diamidino-2-phenylindole; eGFP, enhanced GFP; endo H, endoglycosidase H; IS, inner segment; N, outer nuclear layer; OS, outer segment; PNGase F, peptide-*N*-glycosidase F; RhoKO, rhodopsin KO; S, synapses; TM, transmembrane.

We studied several rhodopsin truncation mutants to understand how regions within the cytoplasmic C terminus of rhodopsin are involved in its proper delivery to the OS. We first expressed FLAG-RhoΔ5, which lacks the OS QVAPA targeting signal and was previously shown to result in mislocalization of rhodopsin from the OS to the rest of the cell body (32). The RhoΔ5 construct was localized throughout the entire rod cell, from the basal synapse to the apical OS, with a mean OS/total intensity value of 0.306 ± 0.155 (Fig. 2*A*). This profile reveals that there is significantly more of the RhoΔ5 construct present outside the OS than a typical untargeted membrane protein (Fig. 1), consistent with previously published data, suggesting that there is a mistargeting signal residing in rhodopsin’s C terminus (324–329 amino acids) (17).

We then expressed a FLAG-RhoΔ25, further truncating the C terminus to the CC anchor, and found this construct was predominantly localized in the OS with a fraction mislocalized to the cell body, showing a mean OS/total intensity value of 0.525 ± 0.126 (Fig. 2*A*). This localization is not significantly different from an untargeted profile, as it is found throughout the cell body and the membrane-rich OS. Finally, we expressed a FLAG-tagged RhoΔ38 that truncates the entire cytoplasmic C terminus, including the amphipathic helix-8. We observed that RhoΔ38 could not reach the OS and appears to be held within internal membranes, likely the ER, as its mean OS/total intensity value was 0.018 ± 0.011 (Fig. 2*A*). Interestingly, each rhodopsin truncation revealed a unique localization profile with RhoΔ5 appearing mistargeted, RhoΔ25 appearing untargeted, and RhoΔ38 appearing ER retained.

It is known that rhodopsin utilizes a conventional secretory pathway through the ER and Golgi (2, 33, 34). We performed deglycosylation experiments to determine whether the trafficking route of our rhodopsin truncations is altered. FLAG-Rho mutants were expressed in RhoKO rods to prevent endogenous rhodopsin from influencing their trafficking pathway. FLAG-Rho constructs were immunoprecipitated and then treated with deglycosylation enzymes before protein mobility was assessed by Western blot. The enzyme peptide-N-glycosidase F (PNGase F) cleaves any type of N-linked glycans, resulting in an increased mobility shift of Rho-FL compared with the untreated control. Endoglycosidase H (endo H) is an enzyme that cleaves unmodified high-mannose N-linked glycans added to proteins in the ER. If the protein reaches the Golgi complex, its N-linked glycan moieties are modified and no longer sensitive to endo H cleavage. We found Rho-FL has a subtle mobility shift upon endo H treatment but is not fully deglycosylated, as shown in the PNGase F-treated sample. This confirms that it traffics through the conventional ER-to-Golgi route (Fig. 2*B*). We also found that RhoΔ5 and RhoΔ25 were sensitive to PNGase F but resistant to endo H, suggesting they use a conventional secretory pathway through the Golgi complex (Fig. 2*B*). In contrast, RhoΔ38 was sensitive to both PNGase F and endo H treatment, suggesting that it does not exit the ER (Fig. 2*B*). Together with our localization data showing that RhoΔ38 cannot reach the OS in WT rods (Fig. 2*A*), we establish that RhoΔ38 does not leave through an unconventional pathway but is retained in the ER.

Previous studies using heterologous expression of rhodopsin in cell culture found that mutations resulting in ER retention can be ubiquitinated and degraded (35, 36, 37). Therefore, we expressed our FLAG-tagged rhodopsin truncation mutants in AD-293 cells and assessed their localization pattern and rate of degradation. We found that FLAG-Rho FL and FLAG-RhoΔ5 were primarily localized to the plasma membrane, where they colocalized with Na/K-ATPase (Fig. S2). FLAG-RhoΔ25 appeared to have a varied localization pattern between the ER and the plasma membrane, suggesting that RhoΔ25 is not processed normally. We observed that FLAG-RhoΔ38 was restricted to the ER, similar to its localization in rod photoreceptors. We then tested protein stability by performing cycloheximide chase experiments. Cycloheximide inhibits translation, preventing the addition of newly synthesized proteins. We did not detect any significant difference in the rate of protein degradation when comparing FLAG-Rho FL to any of the truncated rhodopsin constructs (Fig. S2). Together, these results suggest that truncated rhodopsin constructs are stable even when retained in the ER.

The seven-TM core structure of rhodopsin is known to provide structural integrity and dimerization interfaces, so we wanted to assess the information encoded within the C-terminal region of rhodopsin in isolation. We chose to append rhodopsin’s cytosolic C terminus to a FLAG-tagged, inert, single-pass TM domain from type II activin receptor (38). FLAG-TM-RhoCT behaves like FL rhodopsin with a mean OS/total intensity value of 0.944 ± 0.029 (Fig. 2*C*). Removing the QVAPA OS targeting signal (FLAG-TM-RhoCTΔ5) resulted in a significant mislocalization from the OS to the cell body (mean OS/total intensity value of 0.192 ± 0.041, Fig. 2*C*), supporting the idea that a C-terminal mistargeting signal is present. Further truncation of rhodopsin’s C terminus (FLAG-TM-RhoCTΔ25) increased OS localization, not to FL values, but similar to the untargeted profile (mean OS/total intensity value of 0.688 ± 0.091, Fig. 2*C*). Together, our data show that the targeting information encoded within rhodopsin’s C terminus is conserved whether it is in the context of the core structure of rhodopsin or appended onto an inert TM domain.

### Rhodopsin’s QVAPA targeting signal can alleviate ER retention and restore localization of an untargeted rhodopsin to the OS

**Figure 3:**
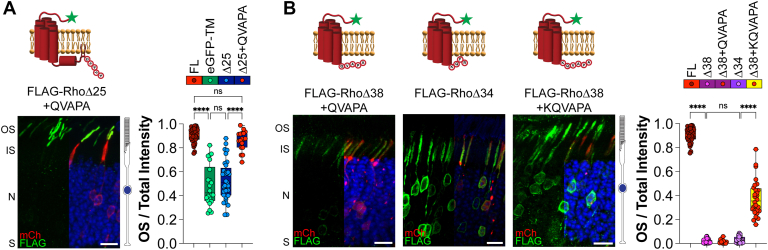
**Rhodopsin’s QVAPA OS targeting signal requires transmembrane stabilization to alleviate ER retention.***A*, WT mouse rods electroporated with FLAG-tagged rhodopsin lacking the final 25 amino acids fused to QVAPA. Δ25 + QVAPA n = 4, 18 images. *B*, WT mouse rods electroporated with the following FLAG-tagged rhodopsin constructs: rhodopsin lacking the final 34 amino acids (RhoΔ34) or lacking the final 38 amino acids fused with either QVAPA (RhoΔ38 + QVAPA) or KQVAPA (RhoΔ38 + KQVAPA). Δ34 n = 6, 31 images; Δ38 + QVAPA n = 7, 32 images; and Δ38 + KQVAPA n = 4, 28 images. Bar graphs show the quotient between the OS signal over the total signal for each construct and plotted next to FLAG-Rho FL or eGFP-TM from Figure 1 or FLAG-RhoΔ25 or FLAG-RhoΔ38 from Figure 2. FLAG staining (*green*), mCherry (*red*) labels transfected rod cells, and DAPI (*blue*) was used to counterstain nuclei. The scale bar represents 10 μm. DAPI, 4′,6-diamidino-2-phenylindole; eGFP, enhanced GFP; ER, endoplasmic reticulum; FL, full length; IS, inner segment; N, outer nuclear layer; OS, outer segment; S, synapses.

We found that RhoΔ25 behaves as an untargeted protein with the same localization profile as an eGFP-TM construct. It was previously shown that appending the C-terminal eight residues of rhodopsin to untargeted constructs drives specific OS localization (22). We appended the minimal QVAPA targeting motif to the RhoΔ25 construct to test whether we could restore OS targeting (FLAG-RhoΔ25 + QVAPA). In WT rods, the addition of the QVAPA sequence specifically targeted RhoΔ25 to the OS so it resembled FL rhodopsin (OS/total intensity value of 0.866 ± 0.052, Fig. 3*A*).

We next wanted to determine whether we could induce normal ER exit of RhoΔ38 by appending the QVAPA targeting sequence to its C terminus (FLAG-RhoΔ38 + QVAPA). In WT rods, FLAG-RhoΔ38 + QVAPA was present in internal membranes surrounding the cell body with no localization to the OSs, similar to FLAG-RhoΔ38 (OS/total intensity value of 0.023 ± 0.016, Fig. 3*B*). Since FLAG-RhoΔ38 lacks the positive lysine residue at the end of the seventh TM helix, the protein topology is likely disrupted, leading to the misfolded phenotype. To circumvent misfolding because of TM instability, we extended the rhodopsin C terminus to FLAG-RhoΔ34, providing the necessary lysine residue to ensure the seventh TM helix is correctly embedded. FLAG-RhoΔ34 was still retained in the ER with an OS/total intensity value of 0.039 ± 0.022 (Fig. 3*B*), suggesting that TM-induced misfolding is not driving ER retention. We then added a lysine residue to the QVAPA targeting signal and appended this onto RhoΔ38 (FLAG-RhoΔ38 + KQVAPA). We found that the OS/total intensity value was significantly increased, suggesting that the KQVAPA alleviated ER retention of RhoΔ38 (OS/total intensity value of 0.365 ± 0.051, Fig. 3*B*). Our data suggest that, in addition to its classical role as an OS targeting signal, the C-terminal QVAPA sequence of rhodopsin can also relieve ER retention if the core helical bundle of rhodopsin is maintained.

### Utilizing RhoKO rods to investigate the OS targeting capacity and elongation properties intrinsic to rhodopsin’s C terminus

**Figure 4:**
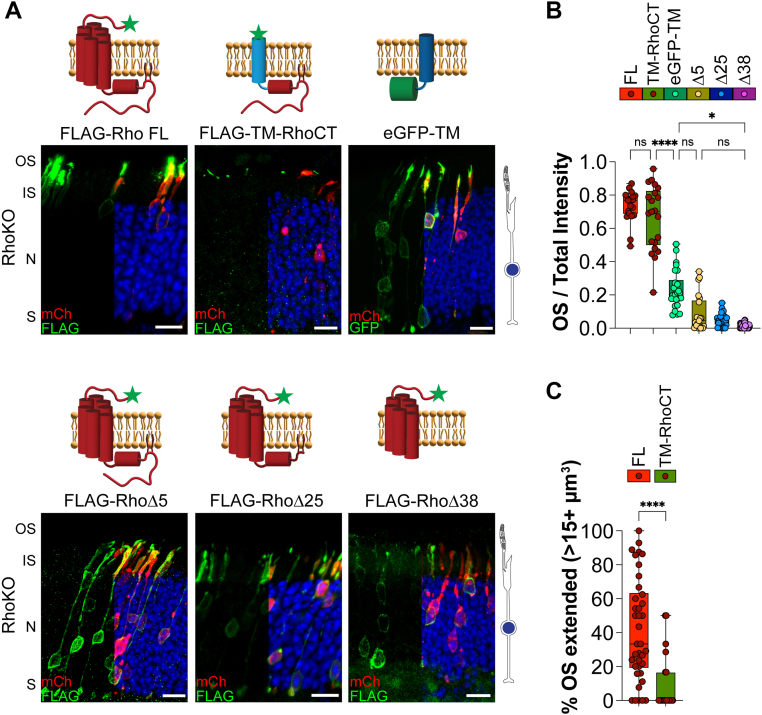
**Rhodopsin cytosolic C terminus is necessary and sufficient for OS targeting but requires its seven-TM core for OS extension in RhoKO rods.***A*, RhoKO mouse rods were electroporated with the eGFP-tagged TM segment 1 from mGluR1 (eGFP-TM) construct or the following FLAG-tagged rhodopsin constructs: full-length rhodopsin (Rho FL), rhodopsin lacking its final five amino acids (RhoΔ5), 25 amino acids (RhoΔ25), or 38 amino acids (RhoΔ38), and the last 38 amino acids of rhodopsin appended to the TM domain of activin (TM-RhoCT). eGFP-TM n = 6, 26 images; FL n = 3, 21 images; Δ5 n = 7, 21 images; Δ25 n = 8, 27 images; Δ38 n = 5, 40 images; and TM-RhoCT n = 5, 21 images. *B*, bar graphs show the quotient between the OS signal over the total signal for each construct as labeled. *C*, graph shows the percentage of extended OSs for RhoKO rods expressing FLAG-Rho FL (FL) or TM-RhoCT; each data point represents a single analyzed Z-stack image. FLAG staining or GFP (*green*), mCherry (*red*) labels transfected rod cells, and DAPI (*blue*) was used to counterstain nuclei. The scale bar represents 10 μm. To the *right* is a *cartoon* of a RhoKO rod photoreceptor, showing disrupted OS structure. DAPI, 4′,6-diamidino-2-phenylindole; eGFP, enhanced GFP; IS, inner segment; N, outer nuclear layer; OS, outer segment; RhoKO, rhodopsin KO; S, synapses; TM, transmembrane.

We expressed our constructs in RhoKO rods to investigate the interplay between rhodopsin’s targeting capacity and its ability to extend the OS. We assessed targeting capacity by quantifying OS/total intensity values for each construct in RhoKO rods. As expected, FLAG-Rho FL expression was specifically localized to rudimentary OSs of RhoKO rods (OS/total intensity value of 0.730 ± 0.036, Fig. 4, *A* and *B*). Similar to FLAG-Rho FL, the FLAG-TM-RhoCT construct was targeted to rudimentary OSs in RhoKO rods (OS/total intensity value for TM-RhoCT 0.654 ± 0.159; Fig. 4, *A* and *B*). Expression of our untargeted membrane reporter, eGFP-TM, resulted in localization throughout the cell body and in the short, rudimentary OSs (OS/total intensity value of 0.227 ± 0.053, Fig. 4, *A* and *B*). The rhodopsin truncation constructs resulted in mislocalization from the rudimentary OSs to the cell body (OS/total intensity values: Δ5 0.107 ± 0.094; Δ25 0.057 ± 0.025; Δ38 0.013 ± 0.008; Fig. 4, *A* and *B*). Together, these results confirm that the C terminus is necessary and sufficient for targeted OS delivery in RhoKO rods.

Qualitatively, we observed that OS structures in RhoKO rods expressing FLAG-Rho FL appear larger than those expressing FLAG-TM-RhoCT (Fig. 4*A*). Fluorescent imaging, however, does not provide structural information about the OS, such as the presence of disc membranes. To quantify and compare FLAG-positive OS volumes in Z-stack images from RhoKO electroporated rods, we developed a 3D Imaris imaging analysis pipeline (see the Experimental procedures section, Fig. S3). From this analysis, we established a threshold between “extended” and “nonextended” OSs in RhoKO rods expressing our FLAG-tagged rhodopsin constructs. We found that the percentage of extended OSs in RhoKO rods expressing FLAG-Rho FL was significantly higher than in rods that expressed FLAG-TM-RhoCT (Fig. 4*C*). Together with our OS/total intensity analysis, we can distinguish between constructs that target the OS compartment and those that also extend the rudimentary OS of RhoKO rods.

### The seven-TM core of rhodopsin is required to extend rudimentary OSs in RhoKO mice

**Figure 5:**
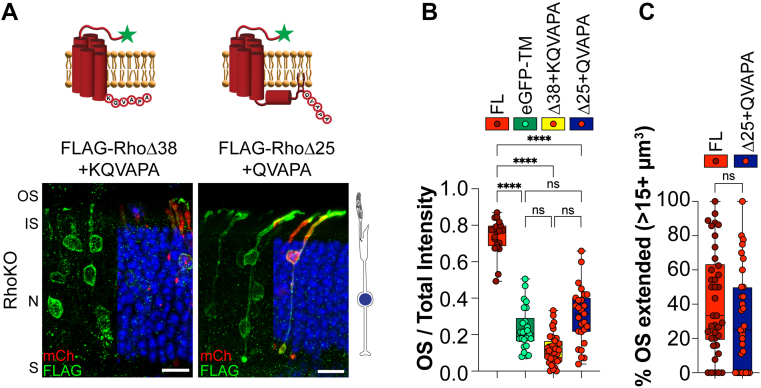
**Rhodopsin requires helix-8 and the CC anchor for proper OS extension of RhoKO rods.***A*, RhoKO mouse rods were electroporated with FLAG-RhoΔ38 + KQVAPA or FLAG-RhoΔ25 + QVAPA. Δ38 + KQVAPA n = 6, 39 images; Δ25 + QVAPA n = 4, 27 images. *B*, bar graphs show the quotient between the OS signal over the total signal for each construct and plotted next to FLAG-Rho FL and untargeted eGFP-TM from Figure 4. *C*, graph shows the percentage of extended OSs in RhoKO rods expressing FLAG-Rho FL (FL) or Δ25 + QVAPA. FLAG staining or GFP (*green*), mCherry (*red*) labels transfected rod cells, and DAPI (*blue*) was used to counterstain nuclei. The scale bar represents 10 μm. To the *right* of the images are *cartoons* of a RhoKO rod photoreceptor. DAPI, 4′,6-diamidino-2-phenylindole; eGFP, enhanced GFP; FL, full length; IS, inner segment; N, outer nuclear layer; OS, outer segment; RhoKO, rhodopsin KO; S, synapses.

We wanted to determine if targeted delivery of rhodopsin's core structure could amass membranes within the rudimentary OSs in RhoKO rods, as we observed with FLAG-Rho FL. We previously found that QVAPA restores OS localization of rhodopsin-truncated constructs in WT mice (Fig. 3*A*), so we electroporated FLAG-RhoΔ38 + KQVAPA or FLAG-RhoΔ25 + QVAPA into RhoKO rods. We found that FLAG-RhoΔ38 + KQVAPA was primarily localized around the cell body, failing to localize to the rudimentary OSs (OS/total intensity value of 0.125 ± 0.045, Fig. 5*A*). The FLAG-RhoΔ25 + QVAPA construct showed more robust localization within the OS compartment; however, its OS/total intensity was not significantly different from the eGFP-TM untargeted membrane construct (OS/total intensity value of 0.307 ± 0.122, Fig. 5*B*). Despite its mislocalization pattern in RhoKO rods, FLAG-RhoΔ25 + QVAPA expression did restore OS volume size similar to FLAG-Rho FL (Fig. 5*C*). This suggests that even though this construct is mislocalized in RhoKO rods, it contains the minimal requirements to extend the rudimentary OS.

**Figure 6:**
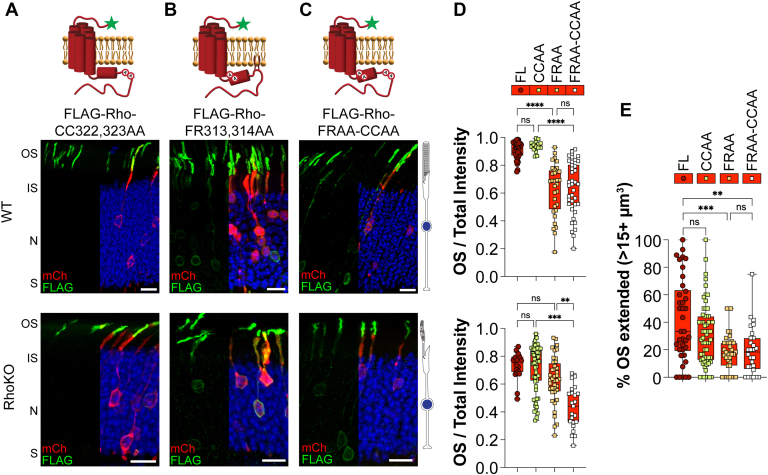
**Rhodopsin mutants that mislocalize in WT rods are unable to accumulate in OSs of RhoKO rods.***A*, FLAG-tagged rhodopsin with a CC(322,323)AA mutation in helix-8, disrupting the palmitoylated cysteine membrane anchor, shows OS targeting comparable to full-length rhodopsin in WT rods (*top panel*). *B*, FLAG-tagged rhodopsin with an FR(313,314)AA mutation in helix-8 displays a mislocalization profile in WT (*top panel*) and RhoKO rods (*bottom panel*). *C*, the double mutant combining FRAA and CCAA mutations phenocopies the FRAA mislocalization profile in WT rods (*top panel*) and runty OS profile in RhoKO rods (*bottom panel*). *D*, bar graphs show the quotient between the OS signal over the total signal for each construct described in *A*–*C*. WT analysis: CCAA n = 4, 15 images; FRAA n = 8, 32 images; and FRAA-CCAA n = 5, 38 images. RhoKO analysis: CCAA n = 9, 61 images; FRAA n = 8, 40 images; and FRAA-CCAA n = 6, 23 images. *E*, the graph shows the percentage of extended OSs in RhoKO rods expressing FLAG-Rho FL (FL), CCAA, FRAA, or FRAA-CCAA. The CCAA mutant elongates the rudimentary OS like FL (*bottom panel*), whereas the FRAA and FRAA-CCAA mutants are unable to sufficiently elongate the OS in RhoKO rods (*bottom panel*). FLAG staining (*green*), mCherry (*red*) labels transfected rod cells, and DAPI (*blue*) was used to counterstain nuclei. The scale bar represents 10 μm. DAPI, 4′,6-diamidino-2-phenylindole; IS, inner segment; N, outer nuclear layer; OS, outer segment; RhoKO, rhodopsin KO; S, synapses.

We examined how rhodopsin helix-8 mutations affect localization and extension. A previous publication found that a palmitoylation-defective rhodopsin mutant resulted in normal rhodopsin localization and OS formation (39). We electroporated a FLAG-Rho-CCAA mutant into WT mice and found it to be properly localized to the OS (OS/total intensity value of 0.936 ± 0.028, Fig. 6, *A* and *D*). And when expressed in RhoKO rods, Rho-CCAA showed a targeted localization in the rudimentary OSs (OS/total intensity value of 0.718 ± 0.131, Fig. 6, *A* and *D*). Analysis of the OS volumes revealed that the FLAG-Rho-CCAA behaved similarly to FLAG-Rho FL in its ability to increase the volume of rudimentary OSs in RhoKO rods (Fig. 6*E*). This result confirms the reliability of our electroporation assay and that rhodopsin palmitoylation is not required for localization or OS elongation, as was previously shown.

The F313 and R314 residues in helix-8 are highly conserved in GPCRs localized to the cilium (40, 41). F313 was shown to form hydrophobic interactions with L68 of TM1, and mutating this phenylalanine to arginine resulted in partial mislocalization of rhodopsin to the inner segment (16). In WT rods, FLAG-Rho-FRAA resulted in a fraction of rhodopsin mislocalized to the cell body (OS/total intensity value of 0.663 ± 0.141, Fig. 6, *B* and *D*), confirming destabilization of the interhelix hydrophobic interaction. We then expressed the FLAG-Rho-FRAA construct in RhoKO rods and found strong localization in rudimentary OSs with a fraction localized to the cell body (Fig. 6*B*). This resulted in FLAG-Rho-FRAA having an OS/total intensity value of 0.664 ± 0.114 (Fig. 6*D*). OS volume analysis from FLAG-Rho-FRAA showed reduced ability to extend rudimentary OSs in RhoKO rods (Fig. 6*E*). Together, our data suggest that destabilizing interhelical hydrophobic interactions prevents sufficient rhodopsin localization to the OS, thereby reducing membrane accumulation and elongation of the compartment in RhoKO rods.

We also tested the combined FRAA and CCAA mutations and found that in WT, it behaved similarly to the FRAA with partial mislocalization from the OS (OS/total intensity value of 0.595 ± 0.157, Fig. 6, *C* and *D*). However, when expressed in RhoKO rods, we found a synergistic effect with increased mislocalization compared with the FRAA mutation (OS/total intensity value of 0.421 ± 0.111, Fig. 6, *C* and *D*). OS volume analysis from the double mutant was not significantly different from the FRAA mutation, suggesting that mislocalized rhodopsin mutants are unable to accumulate OS membranes. We also found that destabilizing the interhelical hydrophobic interactions (FRAA) in the RhoΔ25 truncation, but not the CC anchor (CCAA), showed severe OS localization and volume defects when expressed in WT or RhoKO rods (Fig. S4). Together, our data show that the QVAPA targeting motif and intact helix-8 of rhodopsin are required to amass membranes in the OS.

## Discussion

Our study provides a comprehensive analysis of the localization profile for rhodopsin C-terminal mutants expressed in either WT or RhoKO rods. Analyzing rhodopsin constructs *in vivo* is important, as cell culture experiments do not provide the native environment or trafficking demands unique to rod photoreceptors. We determined whether a given rhodopsin construct is localized to the OS, in the presence or absence of endogenous rhodopsin, and whether the construct can extend the rudimentary OS in RhoKO rods. We found that RhoΔ5, RhoΔ25, and RhoΔ38 truncation constructs each had a distinct localization profile: mistargeted, untargeted, and ER retained, respectively. Despite RhoΔ5 and RhoΔ25 accumulating in the OS of WT rods, we found these constructs did not elongate OS structures in RhoKO rods. In general, localization profiles in RhoKO were hard to compare for these two truncation constructs. While the localization profile for ER-retained RhoΔ38 was significantly different from the untargeted eGFP-TM localization profile (*p* = 0.0141, Fig. 4*B*), both RhoΔ25 and RhoΔ5 fell between these two profiles and were therefore not significantly different from either eGFP-TM or RhoΔ38. Even though we could not categorize these localization patterns, the data suggest that WT OS localization of RhoΔ5 and RhoΔ25 is driven by endogenous rhodopsin, emphasizing that the OS targeting capacity of rhodopsin must be verified in the absence of endogenous rhodopsin.

The disconnect between a construct’s localization pattern in WT and RhoKO was also apparent when we began appending the QVAPA motif onto truncated constructs. By appending the QVAPA targeting signal to RhoΔ25, we fully rescued its localization to the OS in WT rods but not in RhoKO rods. This RhoΔ25 + QVAPA result was surprising since we expected appending the QVAPA signal to the core structure of rhodopsin would result in targeted OS localization in RhoKO rods. Intriguingly, our analysis of OS volumes revealed that even though RhoΔ25 + QVAPA was mislocalized, it was able to extend the rudimentary OSs of RhoKO rods. This was the only construct that performed like this in RhoKO, suggesting that rhodopsin requires an intact seven-TM core, helix 8, CC anchor, and QVAPA targeting signal to elongate the OS compartment.

When we tested a helix-8 disrupting mutation, FRAA, in the RhoΔ25 + QVAPA construct, it resulted in retention in the ER in both WT and RhoKO rods. This suggests that ER retention supersedes targeting, which we also observed to be true when we appended QVAPA onto RhoΔ38, another ER-retained mutant. Only by adding a locking lysine before the QVAPA targeting motif (RhoΔ38 + KQVAPA) could we restore a fraction of the rhodopsin molecules for OS localization in WT rods. However, the presence of the same locking lysine in the absence of QVAPA resulted in an ER retention phenotype (RhoΔ34), which suggests that QVAPA plays a role in supporting ER exit.

Previous studies of ciliary GPCRs have found that the intracellular loop 3 encodes ciliary targeting (42, 43). By examining the C terminus of rhodopsin in isolation (TM-RhoCT), we found similar localization patterns as with rhodopsin’s seven-TM core, suggesting that any additional signals within the core structure of rhodopsin are negligible. Importantly, we also found that the TM-RhoCT construct, while precisely localized to the rudimentary OSs of RhoKO rods, was unable to drive membrane expansion of this compartment.

Our results from mice generally align with what was previously observed in frogs, with one notable difference. In frogs, severe truncations of rhodopsin C terminus (RhoΔ27, RhoΔ32, and RhoΔ38) resulted in predominant localization to the OS (17). This localization pattern was also reported in mice for a GFP-tagged Rho1-314 construct, equivalent to our RhoΔ34 (44). In contrast, we find similar C-terminal rhodopsin truncations (RhoΔ34 and RhoΔ38) to be retained in the ER. A key difference between our study and the previous studies is the fusion of a bulky fluorophore to the C terminus of truncated rhodopsin, whereas we appended a FLAG tag to the N terminus. These fluorophores may provide additional protein stability to the truncated rhodopsin, allowing for ER exit. Regardless of the conflicting results, our study finds that even if RhoΔ38 was able to reach the rod OS in WT rods, shown by appending KQVAPA (Fig. 3), it is unable to generate a proper OS in RhoKO rods (Fig. 5). Thus, our system provides further context for rhodopsin mutants by determining whether they are sufficient to elongate OSs in RhoKO rods. Although we were able to measure volume data from RhoKO rod OSs, one limitation of our study is that we do not examine whether rhodopsin constructs are properly building disc membranes within the OS. This would require ultrastructural analysis, which is beyond the scope of this study.

Finally, our new approach to quantifying OS localization profiles for each electroporated construct across images and multiple animals provides a robust, unbiased way to assess what was previously seen a qualitative results. By performing this analysis, we were able to distinguish the subtle differences between a mistargeting localization pattern and an untargeted “default” localization pattern. Important, as similar experiments previously suggested that rhodopsin’s C terminus lacking the last five amino acids behaves as a “default” membrane protein with no targeting signal (24), which we clearly now see is mistargeted (Fig. 2*C*). Keep in mind, however, that this analysis is tailored for assessing OS localization and is not used to distinguish ER from inner segment plasma membrane. These two membrane compartments are closely aligned in a mouse rod photoreceptor, so they would require super-resolution imaging or a different analysis paradigm to distinguish.

## Experimental procedures

### Animals

Albino CD-1 WT mice were ordered from Charles River Laboratories (strain #022). RhoKO mice were a generous gift from Vadim Arshavsky (Duke University) (28). Mice were handled following protocols approved by the Institutional Animal Care and Use Committees at the University of Michigan (registry number: A3114-01). As there are no known gender-specific differences related to vertebrate photoreceptor development and/or function, male and female mice were randomly allocated to experimental groups. All mice were housed in a 12/12-h light–dark cycle with free access to food and water.

### *In vivo* electroporation of mouse retinas

DNA constructs were electroporated into the retinas of neonatal mice using the protocol as originally described (45), but with modifications as detailed (46). Briefly, P0–P2 mice were anesthetized on ice, had their eyelid and sclera punctured at the periphery of the eye with a 30-gauge needle, and were injected with 0.25 to 0.5 μl of concentrated plasmid in the subretinal space using a blunt-end 32-gauge Hamilton syringe. A plasmid mixture consists of 2 μg/μl of the gene of interest and 1 μg/μl of rhodopsin promoter driving expression of mCherry (pRho:mCherry) as an electroporation marker to identify transfected cells. A complete list of constructs can be found in Table S1. The positive side of a tweezer-type electrode (BTX) was placed over the injected eye, and five 100 V pulses of 50 ms were applied using an ECM830 square pulse generator (BTX). Neonates were returned to their mothers until collection at P20–P22.

### Cell culture

All cell culture experiments were performed using AD-293 cells (Agilent Technologies; catalog no.: 240085; Research Resource Identifier: CVCL_9804). Cells were either plated onto poly-l-lysine glass coverslips (Corning; catalog no.: 354085) in a 24-well plate for imaging or in a 10 cm plate for biochemical assays and grown overnight at 37 °C with 5% CO_2_. Transient transfections were performed 1 day after seeding, and experimental collection was performed 2 days after transfection. The plasmid DNA (0.5 μg/ml) was used for transfections by incubating the DNA with 1 μg/ml polyethyleneimine (Sigma; catalog no.: 408727) in serum-free media for 10 min before being added dropwise to cells that had been changed to serum-free media 1 h prior.

### DNA constructs

A complete list of primers used for cloning can be found in Table S2. For rod-specific expression, complementary DNA sequences were cloned between 5′ AgeI and 3′ NotI cloning sites into a vector driven by a 2.2 kb bovine rhodopsin promoter originally cloned from pRho:DsRed (gift from Dr Connie Cepko; Addgene plasmid #11156; http://n2t.net/addgene:11156). For expression in cell culture, inserts from the pRho plasmids were transferred by molecular cloning with AgeI and NotI cloning sites into a pEGFP-N1 (Clontech) plasmid containing the cytomegalovirus promoter.

### Antibodies

The following commercial primary antibodies were used: mouse monoclonal M2 anti-FLAG (F1804, Sigma–Aldrich); rabbit polyclonal anti-FLAG (F7425, Sigma–Aldrich); goat polyclonal anti-FLAG (ab1257, Abcam); rabbit polyclonal anti-FLAG (PA1-948B, Thermo Fisher); goat polyclonal anti-mCherry (AB0040, OriGene Technologies); rabbit polyclonal anti-Calnexin (C4731, Sigma); and mouse monoclonal anti-Na/K-ATPase (SC58628, Santa Cruz). The following commercial secondary antibodies were used: Donkey anti-mouse AF488 (A21202, Fisher Scientific); Donkey anti-rabbit AF488 (A21206, Fisher Scientific); Donkey anti-sheep AF488 (A11015, Fisher Scientific); Donkey anti-sheep AF568 (A21099, Fisher Scientific); Donkey anti-mouse AF647 (A31571, Fisher Scientific); Donkey anti-Sheep AF647 (A21448, Fisher Scientific); and Donkey anti-Rabbit Peroxidase AffiniPure (711-035-152, Jackson ImmunoResearch).

### Immunofluorescence

Electroporated mouse eyes were enucleated and drop-fixed in 4% paraformaldehyde in 1× PBS (AAJ75889K8, Fisher Scientific) at room temperature for 1 to 2 h before washing with 1× PBS. Eyecups were dissected and embedded in 4% low-gelling temperature agarose in 1× PBS (A9045; Sigma–Aldrich) and cut into 100 μm thick sagittal sections with a vibratome (Leica Biosystems). Free-floating sections were blocked in 5% donkey serum (NC0629457, Thermo Fisher) and 0.5% Triton X-100 (BP151, Fisher Scientific) in 1× PBS for 1 h at room temperature. Then incubated with primary antibodies overnight at 4 °C, washed with 1× PBS, and then incubated in blocking buffer for 1 h at room temperature with secondary antibodies along with 10 μg/ml 4′,6-diamidino-2-phenylindole (102362760, Sigma–Aldrich). Sections were washed in 1× PBS and mounted on slides with ProLong Glass Mounting Media (P36980, Thermo Fisher) and #1.5 coverslips (72204-10, Electron Microscopy Sciences).

Transfected cells on coverslips were fixed in 4% paraformaldehyde for 1 h. Cells were then washed with 1× PBS, permeabilized for 5 min with 0.5% Triton X-100 in 1× PBS, washed with 1× PBS, and nonspecific binding was blocked with a 1-h incubation in 1× PBS containing 0.5% Triton X-100 and 5% normal donkey serum. Coverslips were then incubated overnight at 4 °C with primary antibody diluted in blocking buffer (antibody information listed above), washed in 1× PBS, and incubated for 1 to 2 h at 22 °C with 10 μg/ml 4′,6-diamidino-2-phenylindole and donkey secondary antibodies conjugated with AlexaFluor 488, 568, or 647. Coverslips were washed in 1× PBS and mounted on slides with ImmunoMount for imaging.

Images were acquired at a 1× zoom using a Zeiss Observer 7 inverted microscope equipped with a 63× oil-immersion objective (numerical aperture = 1.40), LSM 800 confocal scanhead outfitted with an Airyscan super-resolution detector controlled by Zen 5.0 software (Carl Zeiss Microscopy). The entire photoreceptor from the OS tip to the synaptic terminal was captured by acquiring 6 to 16 Z-stacks at 1 μm, depending on the angle of the retinal section. Manipulation of images was limited to adjusting the brightness level, image size, rotation, and cropping using FIJI (ImageJ, https://imagej.net/Fiji; ImageJ2, version 2.14.0/1.54f; Build c89e8500e4).

### Image quantification

All imaging processing and analysis for OS/total intensity were performed using the FIJI imaging software. A maximum intensity projection image was produced from a Z-stack image to quantify the ratio of the OS and total fluorescence intensity of the electroporated rod photoreceptors. The maximum stack image was subjected to a 20-pixel rolling-ball background subtraction and threshold set using the Triangle algorithm. When needed, images were subjected to additional manual thresholding to exclude background fluorescence. Binary masks were then produced from the threshold image and fluorescence areas assigned as regions of interest (ROIs). ROIs were then curated to exclude nonspecific staining (*e.g*., secondary antibody binding to blood vessels or immune cells) and generate the total raw integrated density value for a given image. Using the soluble mCherry transfection marker as a guide, an additional “cell body” ROI was added to select the region that contains the inner segment, outer nuclear layer, and synaptic terminals. To calculate the “OS” value, the raw integrated density of the “cell body” ROI was subtracted from the total ROI. For each image, the OS targeting ratio was determined by the resulting quotient values from a simple “OS/total” calculation, where 1.0 is fully targeted and 0.0 is fully excluded from the OS.

OS volume analysis was performed using Imaris software (Ver. 10.2.0, Build 67,164). Imaris surfaces were generated from Z-stack images using automated thresholding or through direct manual selection in the software (Fig. S3*A*). Individual OS volume measurements were then extracted from the generated surfaces. To distinguish an OS as either being “extended” or “nonextended” from the OS volumes, we compared the average OS volumes from FLAG-Rho FL and TM-RhoCT Z-stack images. For TM-RhoCT Z-stack images, this was <15 μm^3^, whereas many FLAG-Rho FL images exceeded this value (Fig. S3*B*). Therefore, we set the “not-extended” OS volume threshold at 15 μm^3^. Using this threshold, we determined the percentage of OSs that were extended (>15 μm^3^) in each Z-stack image. The “%OS extended” value represents the ability of a given construct to extend the rudimentary OS in RhoKO rods.

### Deglycosylation assay of FLAG-rhodopsin constructs

Eyecups from electroporated *Rho*^*−/−*^ mice were collected at P21, screened for positively transfected regions, and pooled into one tube. A minimum of six transfected eyecups were used for each immunoprecipitation/deglycosylation assay. Eyecups were lysed in 1 ml 1% *n*-dodecyl β-d-maltoside in PBS with protease inhibitors. Lysates were cleared at 15,000 rpm for 10 min at 4 °C, and supernatant was collected for FLAG immunoprecipitation. FLAG-Rho FL n = 3; FLAG-RhoΔ5 n = 2; FLAG-RhoΔ25 n = 3; and FLAG-RhoΔ38 n = 2.

For PNGase F treatment, the lysate is combined with 2 μl each glycoprotein denaturing buffer, GlycoBuffer 2, 10% NP-40, 1 μl PNGase F (P0708L, New England Biolabs), and H_2_O as necessary to a final volume of 20 μl. The reaction was incubated for 1 h at 37 °C. For endo H (P0702L, New England Biolabs) treatment, the same procedure was followed, except that GlycoBuffer 3 was used instead of GlycoBuffer 2. For control experiments, reactions used GlycoBuffer 2 without enzymes. The reaction was terminated by cooling on ice or freezing at −20 °C. The ×sample buffer (5x) with 100 mM DTT was added to each sample. Western blotting was performed by running lysates on SDS-PAGE using AnykD Criterion TGX Precast Midi Protein Gels (5671124, Bio-Rad) was followed by transfer at 66 mV for 120 min onto Immun-Blot Low Fluorescence Polyvinylidene Difluoride Membrane (1620264, Bio-Rad). Membranes were blocked using Intercept Blocking Buffer (927-70003; LI-COR Biosciences). Primary antibodies were diluted in 50%/50% of Intercept/PBS with Tween-20 (PBST) and incubated overnight, rotating at 4 °C. The next day, membranes were rinsed three times with PBST before incubating in the corresponding secondary donkey antibodies conjugated with AlexaFluor 680 or 800 (LiCor Bioscience) in 50%/50%/0.02% of intercept/PBST/SDS for 2 h at 4 °C. Bands were visualized and quantified using the Odyssey CLx Infrared Imaging System (LiCor Bioscience). Uncropped Western blot images can be found in Figure S5.

### Experimental design and statistical analyses

All *in vivo* electroporated mouse retinas were analyzed at postnatal day 20 to 22. Biological replicates (n’s) were defined as a positively expressing eye, with a minimum of three n’s analyzed for every DNA construct. Each “n” consisted of multiple Z-stack images within the expressing retinal tissue. Animals of both sexes were used for all experimental models and were not distinguished in the data. Statistical analysis was performed using Prism 10.0 (GraphPad Prism Software, version 10.4.2; GraphPad Software, Inc). *p* Values for the OS targeting quotients were determined by using the mean quotient value from each individual “n” (average of averages). Constructs in either WT or RhoKO mice were compared using a one-way ANOVA (Tukey’s test to correct for multiple comparisons). All *p* values can be found in the multicomparison graphs provided in Figure S1. *p* Values for the OS volume data were also determined using one-way ANOVA.

## Data availability

All the data are contained within this article.

## Supporting information

This article contains supporting information.

## Conflict of interest

The authors declare that they have no conflicts of interest with the contents of this article.

## References

[1] Gulati S., Palczewski K. (2023). Structural view of G protein-coupled receptor signaling in the retinal rod outer segment. Trends Biochem. Sci..

[2] Deretic D., Papermaster D.S. (1991). Polarized sorting of rhodopsin on Post-Golgi membranes in frog retinal photoreceptor cells. J. Cell Biol..

[3] Bok D., Young R.W. (1972). The renewal of diffusely distributed protein in the outer segments of rods and cones. Vision Res..

[4] Haggerty K.N., Eshelman S.C., Sexton L.A., Frimpong E., Rogers L.M., Agosto M.A., Robichaux M.A. (2024). Super-resolution mapping in rod photoreceptors identifies rhodopsin trafficking through the inner segment plasma membrane as an essential subcellular pathway. PLoS Biol..

[5] Hekmatara M., Thompson S.L., Haggerty K.N., Hagen S., Brothers B.A., Daniels B. (2025). Super-resolution microscopy reveals a Rab6a-dependent trafficking hub for rhodopsin at the mammalian rod photoreceptor Golgi. Biol. Open.

[6] Hartong D.T., Berson E.L., Dryja T.P. (2006). Retinitis pigmentosa. Lancet.

[7] Athanasiou D., Aguila M., Bellingham J., Li W., McCulley C., Reeves P.J., Cheetham M.E. (2018). The molecular and cellular basis of rhodopsin retinitis pigmentosa reveals potential strategies for therapy. Prog. Retin. Eye Res..

[8] Audo I., Manes G., Mohand-Saïd S., Friedrich A., Lancelot M.E., Antonio A., Moskova-Doumanova V. (2010). Spectrum of rhodopsin mutations in French autosomal dominant rod-cone dystrophy patients. Invest Ophthalmol. Vis. Sci..

[9] Cideciyan A.V., Hood D.C., Huang Y., Banin E., Li Z.Y., Stone E.M., Milam A.H., Jacobson S.G. (1998). Disease sequence from mutant rhodopsin allele to rod and cone photoreceptor degeneration in man. Proc. Natl. Acad. Sci. U.S.A..

[10] Thompson S.L., Crowder S.M., Hekmatara M., Sechrest E.R., Deng W.T., Robichaux M.A. (2025). P23H rhodopsin accumulation causes transient disruptions to synaptic protein levels in rod photoreceptors in a model of retinitis pigmentosa. Dis. Model Mech..

[11] O'Brien P.J. (1982). Glycosylation of rhodopsin. Methods Enzymol..

[12] Fukuda M.N., Papermaster D.S., Hargrave P.A. (1982). Structural analysis of carbohydrate moiety of bovine rhodopsin. Methods Enzymol..

[13] Palczewski K., Kumasaka T., Hori T., Behnke C.A., Motoshima H., Fox B.A., Le Trong I. (2000). Crystal structure of rhodopsin: a G protein-coupled receptor. Science.

[14] Burns M.E., Arshavsky V.Y. (2005). Beyond counting photons: trials and trends in vertebrate visual transduction. Neuron.

[15] Weiss E.R., Osawa S., Shi W., Dickerson C.D. (1994). Effects of carboxyl-terminal truncation on the stability and G protein-coupling activity of bovine rhodopsin. Biochemistry.

[16] Verma D.K., Malhotra H., Woellert T., Calvert P.D. (2023). Hydrophobic interaction between the TM1 and H8 is essential for rhodopsin trafficking to vertebrate photoreceptor outer segments. J. Biol. Chem..

[17] Lodowski K.H., Lee R., Ropelewski P., Nemet I., Tian G., Imanishi Y. (2013). Signals governing the trafficking and mistrafficking of a ciliary GPCR, rhodopsin. J. Neurosci..

[18] Chen Q., Plasencia M., Li Z., Mukherjee S., Patra D., Chen C.-L., Klose T. (2021). Structures of rhodopsin in complex with G-protein-coupled receptor kinase 1. Nature.

[19] Kang Y., Zhou X.E., Gao X., He Y., Liu W., Ishchenko A., Barty A. (2015). Crystal structure of rhodopsin bound to arrestin by femtosecond X-ray laser. Nature.

[20] Zhou X.E., He Y., de Waal P.W., Gao X., Kang Y., Van Eps N., Yin Y. (2017). Identification of phosphorylation codes for arrestin recruitment by G protein-coupled receptors. Cell.

[21] Sung C.H., Makino C., Baylor D., Nathans J. (1994). A rhodopsin gene mutation responsible for autosomal dominant retinitis pigmentosa results in a protein that is defective in localization to the photoreceptor outer segment. J. Neurosci..

[22] Tam B.M., Moritz O.L., Hurd L.B., Papermaster D.S. (2000). Identification of an outer segment targeting signal in the COOH terminus of rhodopsin using transgenic Xenopus laevis. J. Cell Biol..

[23] Baker S.A., Haeri M., Yoo P., Gospe S.M., Skiba N.P., Knox B.E., Arshavsky V.Y. (2008). The outer segment serves as a default destination for the trafficking of membrane proteins in photoreceptors. J. Cell Biol..

[24] Pearring J.N., Salinas R.Y., Baker S.A., Arshavsky V.Y. (2013). Protein sorting, targeting and trafficking in photoreceptor cells. Prog. Retin. Eye Res..

[25] Pearring J.N., Lieu E.C., Winter J.R., Baker S.A., Arshavsky V.Y. (2014). R9AP targeting to rod outer segments is independent of rhodopsin and is guided by the SNARE homology domain. Mol. Biol. Cell.

[26] Young R.W. (1967). The renewal of photoreceptor cell outer segments. J. Cell Biol..

[27] Besharse J.C., Hollyfield J.G. (1979). Turnover of mouse photoreceptor outer segments in constant light and darkness. Invest. Ophthalmol. Vis. Sci..

[28] Lem J., Krasnoperova N.V., Calvert P.D., Kosaras B., Cameron D.A., Nicolò M., Makino C.L., Sidman R.L. (1999). Morphological, physiological, and biochemical changes in rhodopsin knockout mice. Proc. Natl. Acad. Sci. U.S.A..

[29] Lee E.S., Burnside B., Flannery J.G. (2006). Characterization of peripherin/rds and rom-1 transport in rod photoreceptors of transgenic and knockout animals. Invest Ophthalmol. Vis. Sci..

[30] Humphries M.M., Rancourt D., Farrar G.J., Kenna P., Hazel M., Bush R.A., Sieving P.A. (1997). Retinopathy induced in mice by targeted disruption of the rhodopsin gene. Nat. Genet..

[31] Pearring J.N., Spencer W.J., Lieu E.C., Arshavsky V.Y. (2015). Guanylate cyclase 1 relies on rhodopsin for intracellular stability and ciliary trafficking. eLife.

[32] Concepcion F., Chen J. (2010). Q344ter mutation causes mislocalization of rhodopsin molecules that are catalytically active: a mouse model of Q344ter-induced retinal degeneration. PLoS One.

[33] Tian G., Ropelewski P., Nemet I., Lee R., Lodowski K.H., Imanishi Y. (2014). An unconventional secretory pathway mediates the cilia targeting of peripherin/rds. J. Neurosci..

[34] Pearring J.N., Martínez-Márquez J.Y., Willer J.R., Lieu E.C., Salinas R.Y., Arshavsky V.Y. (2021). The GARP domain of the Rod CNG Channel’s β1-Subunit contains distinct sites for outer segment targeting and connecting to the photoreceptor disk rim. J. Neurosci..

[35] Illing M.E., Rajan R.S., Bence N.F., Kopito R.R. (2002). A rhodopsin mutant linked to autosomal dominant retinitis pigmentosa is prone to aggregate and interacts with the ubiquitin proteasome system. J. Biol. Chem..

[36] Wan A., Place E., Pierce E.A., Comander J. (2019). Characterizing variants of unknown significance in rhodopsin: a functional genomics approach. Hum. Mutat..

[37] Chiang W.C., Kroeger H., Sakami S., Messah C., Yasumura D., Matthes M.T., Coppinger J.A. (2015). Robust endoplasmic reticulum-associated degradation of rhodopsin precedes retinal degeneration. Mol. Neurobiol..

[38] Gospe S.M., Baker S.A., Kessler C., Brucato M.F., Winter J.R., Burns M.E., Arshavsky V.Y. (2011). Membrane attachment is key to protecting transducin GTPase-activating complex from intracellular proteolysis in photoreceptors. J. Neurosci..

[39] Wang Z., Wen X.H., Ablonczy Z., Crouch R.K., Makino C.L., Lem J. (2005). Enhanced shutoff of phototransduction in transgenic mice expressing palmitoylation-deficient rhodopsin. J. Biol. Chem..

[40] Yang S., Bahl K., Chou H.T., Woodsmith J., Stelzl U., Walz T., Nachury M.V. (2020). Near-atomic structures of the BBSome reveal the basis for BBSome activation and binding to GPCR cargoes. Elife.

[41] Klink B.U., Zent E., Juneja P., Kuhlee A., Raunser S., Wittinghofer A. (2017). A recombinant BBSome core complex and how it interacts with ciliary cargo. Elife.

[42] Berbari N.F., Johnson A.D., Lewis J.S., Askwith C.C., Mykytyn K. (2008). Identification of ciliary localization sequences within the third intracellular loop of G protein-coupled receptors. Mol. Biol. Cell.

[43] Barbeito P., Tachibana Y., Martin-Morales R., Moreno P., Mykytyn K., Kobayashi T., Garcia-Gonzalo F.R. (2021). HTR6 and SSTR3 ciliary targeting relies on both IC3 loops and C-terminal tails. Life Sci. Alliance.

[44] Chadha A., Paniagua A.E., Williams D.S. (2021). Comparison of ciliary targeting of two rhodopsin-like GPCRs: role of C-Terminal localization sequences in relation to cilium type. J. Neurosci..

[45] Matsuda T., Cepko C.L. (2004). Electroporation and RNA interference in the rodent retina in vivo and in vitro. Proc. Natl. Acad. Sci. U.S.A.

[46] Salinas R.Y., Pearring J.N., Ding J.D., Spencer W.J., Hao Y., Arshavsky V.Y. (2017). Photoreceptor discs form through peripherin-dependent suppression of ciliary ectosome release. J. Cell Biol..

