# Supplementary material for: Targeted delivery of rhodopsin’s assembled core is required for outer segment extension in mouse rod photoreceptors
Source: J Biol Chem. 2025 Dec 23;302(2):111106. doi: 10.1016/j.jbc.2025.111106 (PMC12816902; doi:10.1016/j.jbc.2025.111106)
Supplement: Supporting information [file mmc1.pdf]

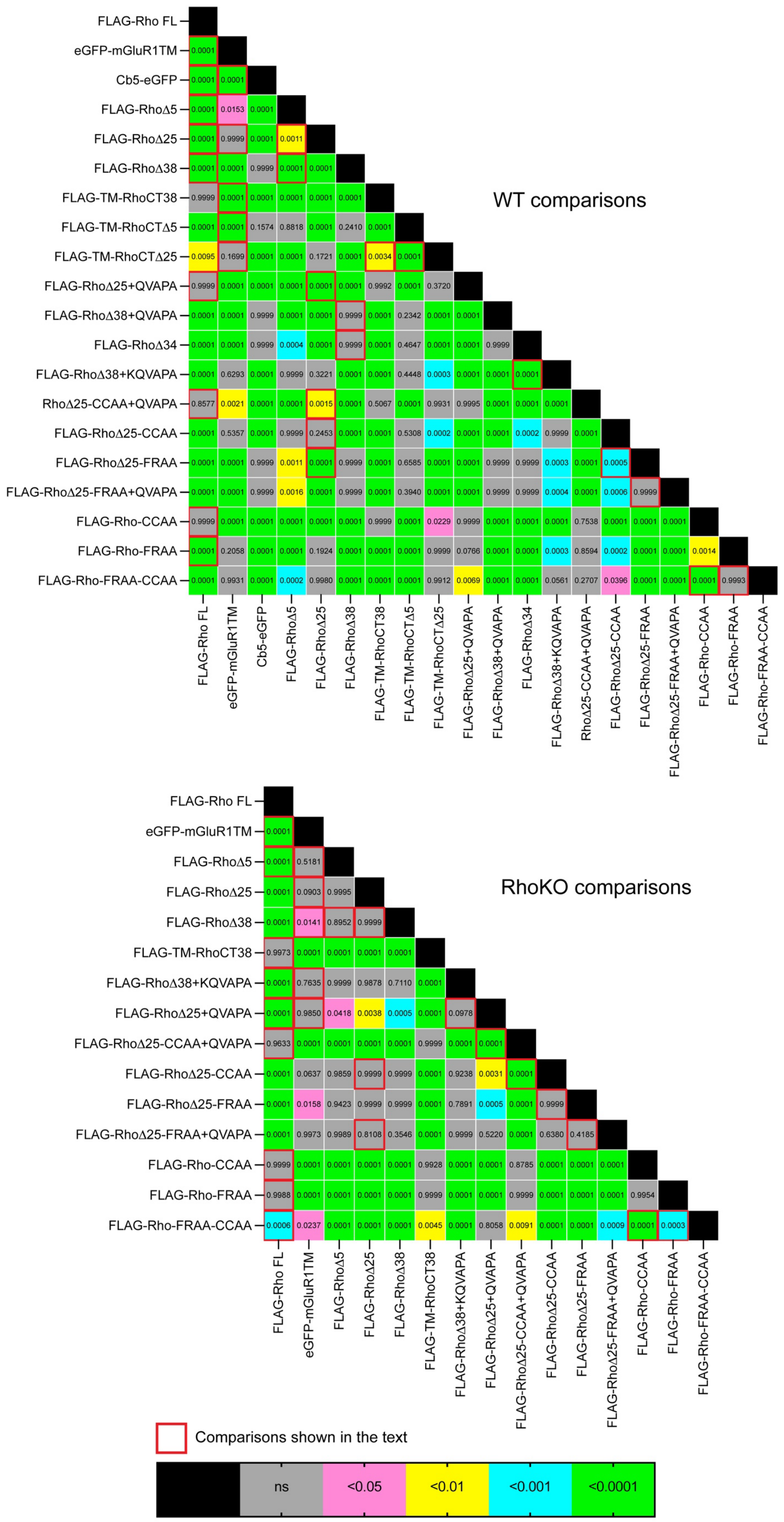


**Figure S1: One-way ANOVA p-Value comparisons grids.** Each construct tested for OS/Total Intensity in this study was compared to every other construct expressed in WT or RhoKO rods. Red boxes highlight the p-Values shown in the figures and results. Heat-map legend shows significance values from one-way ANOVA comparisons.

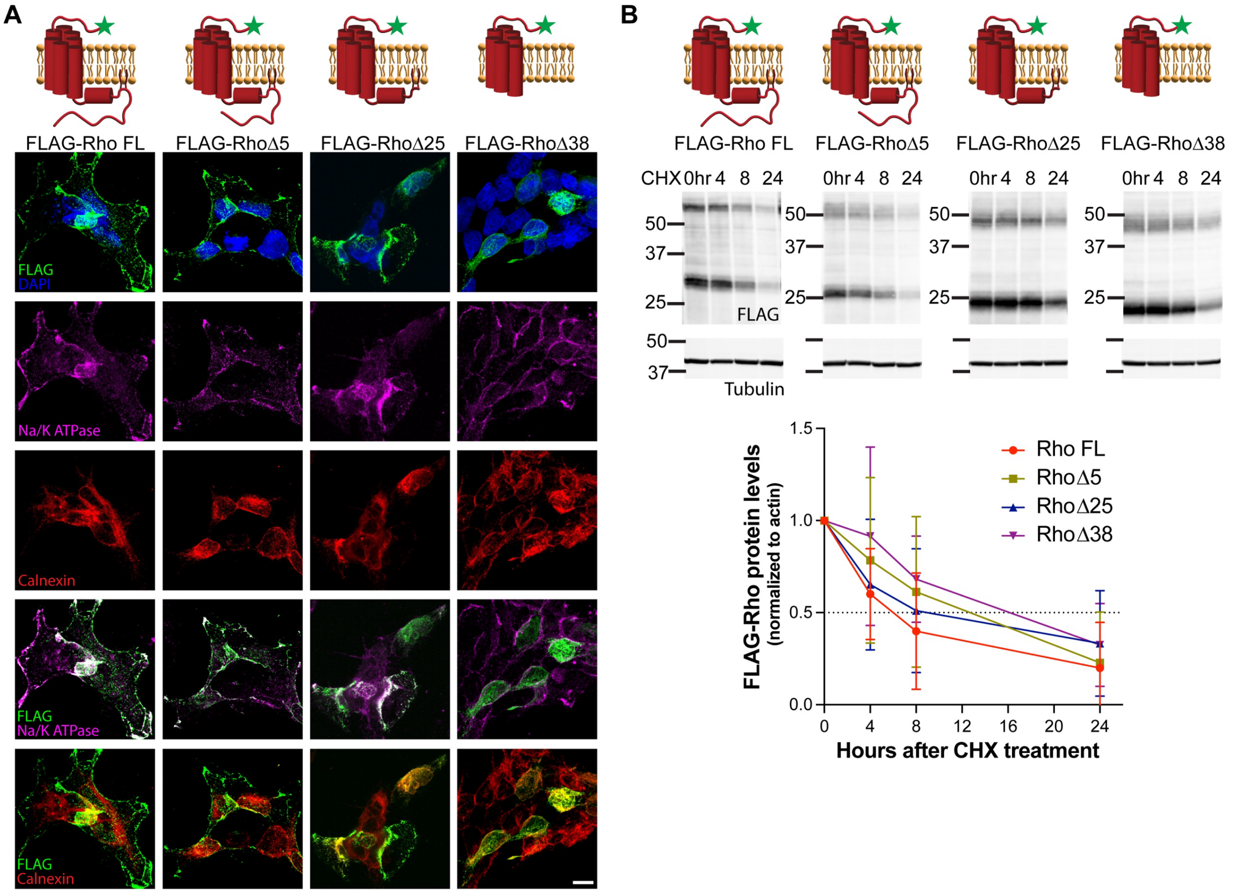


**Figure S2: Rhodopsin truncations expressed in AD293 cells show varied localization patterns with no change to their rates of degradation. A**. FLAG-tagged rhodopsin constructs transfected into AD293 cells (green). Counterstained with DAPI (blue) to mark nuclei, anti-Na/K ATPase antibody (magenta) to mark the plasma membrane, or anti-calnexin antibody (red) to mark the ER. FLAG and Na/K ATPase co-localization shown in white, FLAG and calnexin co-localization shown in yellow. Scale Bar, 10 µm. **B.** Lysates from AD293 cells transfected with FLAG-tagged rhodopsin constructs were collected before and after 4, 8 or 24hr treatment with cycloheximide (CHX) and analyzed by Western blot. We observe monomeric FLAG-Rho variants run at ~25 kDa, with dimers running at ~50 kDa. CHX-treatment pauses protein synthesis and enables the analysis of degradation rates over time. Line graph analyzing level of monomer FLAG-Rho variants normalized to actin over time is shown below. Degradation rates between the tested constructs were not significant.

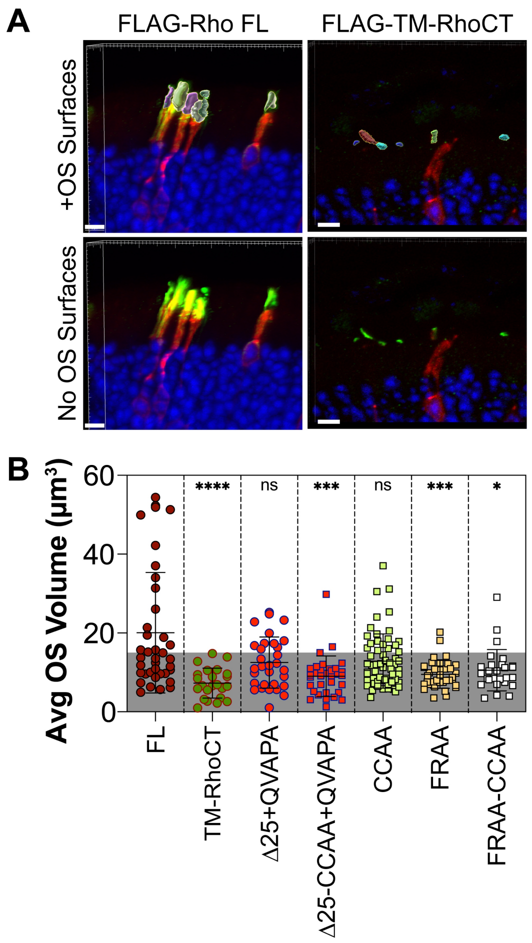


**Figure S3: Full data set of constructs tested for OS volume in transfected RhoKO rods. A**. Example images of transfected RhoKO rods with (top row) or without (bottom row) overlayed outer segment volume surfaces generated using the Imaris image analysis software. Scale bar, 5 μm. **B.** Graph shows the outer segment volume values for RhoKO rods expressing constructs with apparent OS extension. Note that only Rho FL, RhoΔ25+QVAPA and Rho-CCAA were found to extend RhoKO rudimentary outer segments. Each data point represents the average of the outer segment volumes in a single image. Grey area represents the threshold value used to assign rudimentary outer segments from RhoKO rods extended; TM-RhoCT was used to determine this threshold.

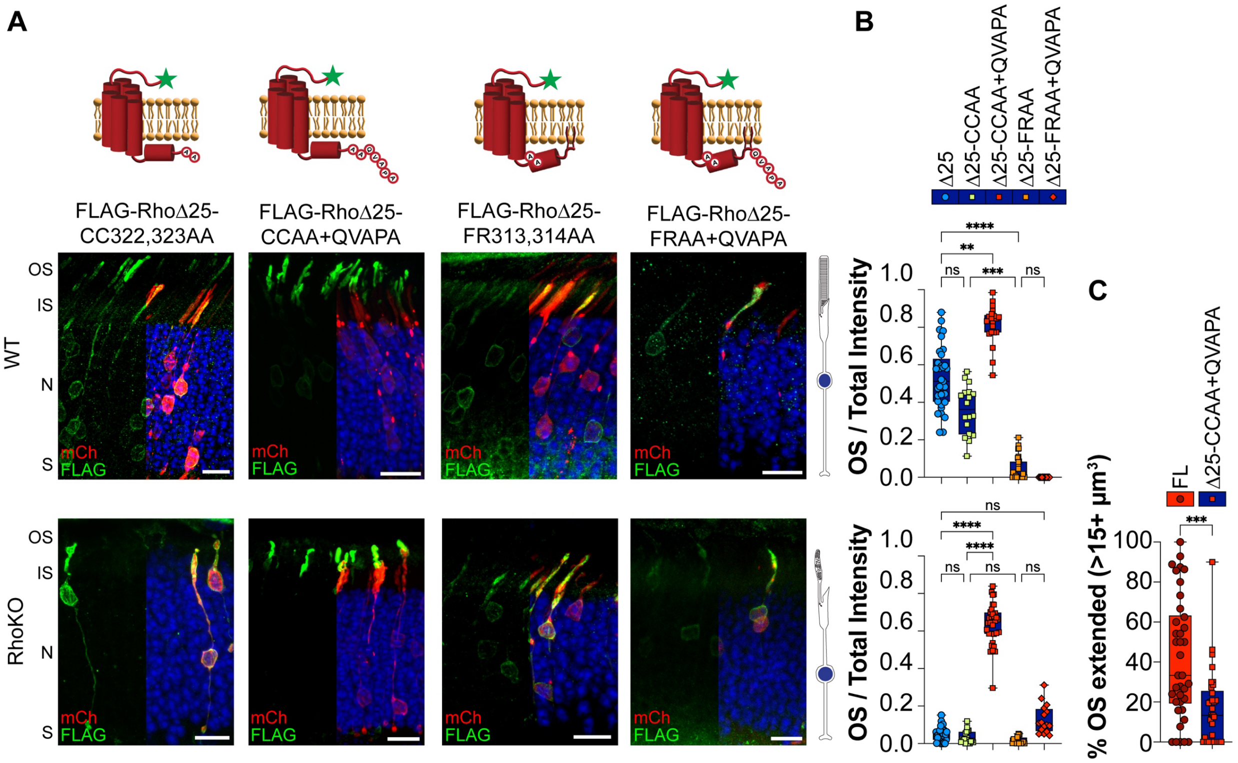


**Figure S4: Disruption of helix-8 in RhoΔ25, but not CC anchors, results in failure to exit the ER. A.** WT or RhoKO mouse rods (top or bottom panels, respectively) electroporated with FLAG-tagged rhodopsin lacking the final 25 amino acids (RhoΔ25) with the following modifications: CC anchor mutated to alanines (CCAA), CCAA mutation with C-terminally appended QVAPA (CCAA+QVAPA), FR motif residues mutated to alanines (FRAA), or FRAA mutation with C-terminally appended QVAPA (FRAA+QVAPA). **B.** Bar graphs show the quotient between the outer segment signal over the total signal for each construct described. WT analysis: Δ25-CCAA n=4, 18 images; Δ25-CCAA+QVAPA n=6, 33 images; Δ25-FRAA n=6, 28 images; Δ25-FRAA+QVAPA n=3, 15 images. RhoKO analysis: Δ25-CCAA n=4, 19 images; Δ25-CCAA+QVAPA n=6, 34 images; Δ25-FRAA n=4, 16 images; Δ25-FRAA+QVAPA n=6, 16 images. **C.** Graph shows the percentage of extended outer segments for RhoKO rods expressing Flag-Rho FL (FL) or Δ25-CCAA+QVAPA. FLAG staining (green), mCherry (red) labels transfected rod cells, and DAPI (blue) used to counter-stain nuclei. Scale Bar, 10 μm.

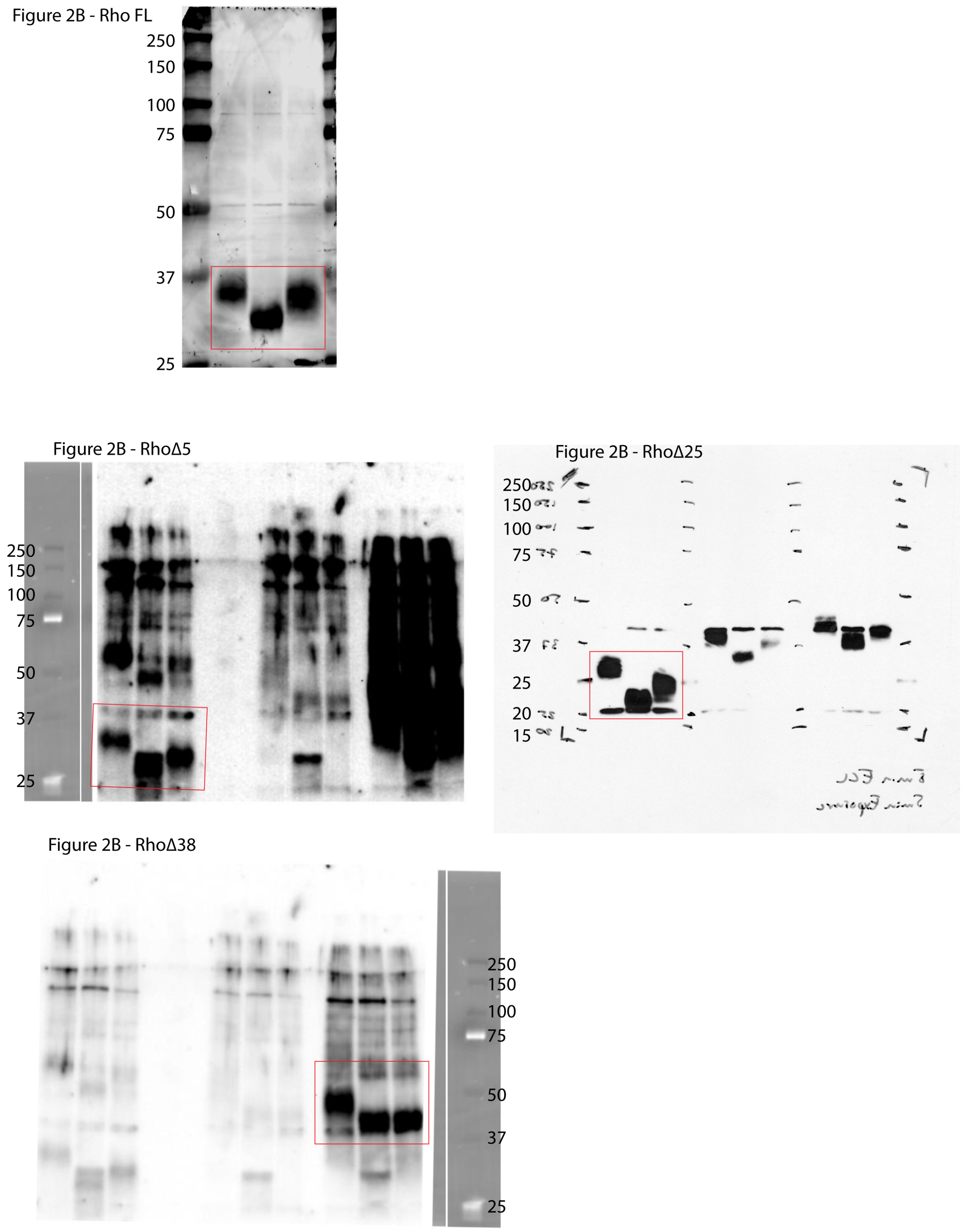


**Figure S5: Uncropped images of Western blots used in Figure 2B.**

| Construct | Localization in WT | | OS extension in RhoKO | Figure |
|---|---|---|---|---|
| | OS | IS | | |
| FLAG-Rho-FL | + | - | + | Fig. 1, Fig. 4, Sup Fig. 2 and Sup Fig. 4 |
| EGFP-TM | + | + | NA | Fig. 1 and Fig. 4 |
| EGFP-Cb5-TM | - | + | NA | Fig. 1 |
| FLAG-RhoΔ5 | + | + | - | Fig. 2, Fig. 4 and Sup Fig. 2 |
| FLAG-RhoΔ25 | + | + | - | Fig. 2, Fig. 4 and Sup Fig. 2 |
| FLAG-RhoΔ38 | - | + | - | Fig. 2, Fig. 4 and Sup Fig. 2 |
| FLAG-TM-RhoCT | + | - | - | Fig. 2, Fig. 4 and Sup Fig. 4 |
| FLAG-TM-RhoCTΔ5 | + | + | NA | Fig. 2 |
| FLAG-TM-RhoCTΔ25 | + | + | NA | Fig. 2 |
| FLAG-RhoΔ25+QVAPA | + | - | + | Fig. 3, Fig. 5 and Sup Fig. 3 |
| FLAG-RhoΔ38+QVAPA | - | + | - | Fig. 3 |
| FLAG-RhoΔ34 | - | + | NA | Fig. 3 |
| FLAG-RhoΔ38+KQVAPA | + | + | - | Fig. 3 and Fig. 5 |
| FLAG-RhoΔ25-CCAA+QVAPA | + | - | - | Sup Fig. 3 and Sup Fig. 4 |
| FLAG-RhoΔ25-CCAA | + | + | - | Sup Fig. 3 and Sup Fig. 4 |
| FLAG-RhoΔ25-FRAA | - | + | - | Sup Fig. 3 |
| FLAG-RhoΔ25-FRAA+QVAPA | - | + | - | Sup Fig. 3 |
| FLAG-Rho-CC322,323AA | + | - | + | Fig. 6, Sup Fig. 3 and Sup Fig. 4 |
| FLAG-Rho-FR313,314AA | + | + | - | Fig. 6, Sup Fig. 3 and Sup Fig. 4 |
| FLAG-Rho-FRAA-CCAA | + | + | - | Fig. 6, Sup Fig. 3 and Sup Fig. 4 |

"+" = attribute observed; "-" = attribute not observed
NA = not tested

**Table S1: Summary of all transgenic constructs presented in this study.**

| Primer Name | Sequence (5' --> 3') | Usage |
|---|---|---|
| pRho2.2K fwd seq | CGCCGCCGGGGATCCTCTAG | Amplify upstream from pRho-Rho; pRho sequencing |
| pRho2.2K rev seq | CCAGCCACCACCTTCTGATAG | Amplify downstream from pRho-Rho; pRho sequencing |
| Rho-Nterm-seq-R | TTCCGCCAGGTAGTACTGC | Sequencing mouse Rho |
| Rho-Mid-seqR | GATTCGTTGTTGACCTCAGG | Sequencing mouse Rho |
| SABRhoCtd5-NotI-BamHI-Rev | GCATGGATCCGCGGCCGCTTAGCTGGTCT CCGTCTTGG | Rhodopsin $\Delta$5 truncation |
| Rho-D25-NotI-rev | ATGAGCGGCCGCTTAGCAGCACAGCGTGG TGAG | Rhodopsin $\Delta$25 truncation |
| Rho1-314_NotI Rev | GCATGCGGCCGCTTACCGGAACTGCTTGTT CAAC | Rhodopsin $\Delta$34 truncation |
| RhoD38_Rev | GCGGCCGCTCAGTTCAACATGATGTAGATG ACC | Rhodopsin $\Delta$38 truncation after $7^{th}$ TMD |
| RhoF313A_Fwd | CATGTTGAACAAGCAGGCCCGGAACTGTAT GCTCACC | Mutagenesis primer for F313A mutant |
| RhoF313A_Rev | GGTGAGCATACAGTTCCGGGCCTGCTTGTT CAACATG | Mutagenesis primer for F313A mutant |
| RhoR314A_Fwd | CATGTTGAACAAGCAGTTCGCGAACTGTAT GCTCACCACG | Mutagenesis primer for R314A mutant |
| RhoR314A_Rev | CGTGGTGAGCATACAGTTCGCGAACTGCTT GTTCAACATG | Mutagenesis primer for R314A mutant |
| NotI-+QVXPX-RhoD25_Rev | GCATGCGGCCGCTCAGGCTGGAGCCACCT GGCAGCACAGCGTGGTGAGCATAC | To append QVAPA motif to $\Delta$25 constructs |
| Rho-CC322,323AA _Fwd | CTGTATGCTCACCACGCTGGCCGCCGGCA AGAATCCACT | Mutagenesis primer for CC322, 323AA |
| Rho-CC322,323AA _Rev | CAGTGGATTCTTGCCGGCGGCCAGCGTGG TGAGCATACA | Mutagenesis primer for CC322, 323AA |
| NotI-RhoD25-CCAA_Rev | GCATGCGGCCGCTCAGGCGGCCAGCGTGG TGAGCATACAG | Rhodopsin $\Delta$25 truncation with CC322,323AA mutation |
| NotI-+QVXPX-RhoD25-CCAA_R | GCATGCGGCCGCTCAGGCTGGAGCCACCT GGGCGGCCAGCGTGGTGAGCATAC | $\Delta$25 truncation with CCAA mutation and QVAPA appended |
| NotI-+QVAPA-RhoD38_Rev | GCATGCGGCCGCTCAGGCTGGAGCCACCT GGTTCAACATGATGTAGATGACC | Rhodopsin $\Delta$38 truncation with QVAPA appended |
| NotI-+KQVAPA-RhoD38_Rev | GCATGCGGCCGCTCAGGCTGGAGCCACCT GCTTGTTCAACATGATGTAGATGA | Rhodopsin $\Delta$38 truncation with KQVAPA appended |
| Flag-ActTM-Fwd | GACTACAAGGACGACGATGACAAGAGATCA TTTCCGGAGATG | FLAG-tagged TMD fused to Rhodopsin C-termini |
| ActSS-Flag-Rev | CTTGTCATCGTCGTCCTTGTAGTCGCCAAG TATAGCACCTG | FLAG-tagged TMD fused to Rhodopsin C-termini |

**Table S2: Oligonucleotide primers to generate and sequence confirm constructs used in this study.**
